# Medical and Philosophical News

**Published:** 1781-05

**Authors:** 


At the ſale of the late Mr. Blackall's anatomical preparations on Monday, May 1, the double uterus and vagina deſcribed by Dr. Purcell of Dublin in the 64th volume of the *Philoſ. Tranſ.* was purchaſed by Mr. John Hunter for fifty guineas.—In the above collection, among ſeveral preparations of diſeaſes, the following, on account count of their ſingularity, ſeem worthy of being recorded. 1. *A tumour compreſſing the oeſophagus.*—This was taken from a man who died in St. Bartholomew's Hoſpital in Dec. 1779, and whoſe deglutition was prevented by a large tumour ſituated in the lungs, below the bifurcation of the trachea. During life, a tumour (ſeemingly ſcrophulous) appeared on the left ſide of the neck above the level of the larynx. When cut into after death, it was found to contain a fœtid, conſiſtent matter. The pharynx and upper part of the oeſophagus were overſpread with a malignant (apparently cancerous) ulceration, which had deſtroyed in one point the ſubſtance of the oeſophagus, and there the cavity of the tumour in which the matter before-mentioned was lodged, communicated with the oeſophagus. 2. *A diſeaſed hand.*—This was amputated on account of a caries of the bones of the middle finger, and an hæmorrhage which could not be reſtrained. On diſſection, all the arteries of the part appeared diſeaſed, dilated, and aneuriſmal. 3. *A communication between the ſtomach and gall bladder.*—This was taken from a man who died of an aſcites, after being tapped. On diſſection, a conſiderable quantity of blood was found in the abdomen. 4. *A communication between the*

*trachea*

*trachea and the pulmonary artery.*—The ſubject of this diſeaſe died in St. Bartholomew's hoſpital in 1780, by a ſudden diſcharge of blood, ſeemingly arterial, from the mouth. On diſſection, a large communication appeared to have taken place between the right branch of the pulmonary artery, and the ſame branch of the trachea.

5. *A diſeaſe of the urethra and bladder.*—This preparation was taken from a man whoſe complaints originated from an obſtructed urethra; for ſome time before his death, his urine, inſtead of paſſing through the penis, was diſcharged through five or ſix fiſtulæ in the ſcrotum, the patient experiencing great pain about the bladder and rectum. On diſſection, oppoſite to the fiſtulæ in the ſcrotum, an obſtruction was diſcovered in the urethra, occaſioned primarily by a ſtricture of that canal, but completed by ſeven or eight calculi which had been cauſed by it, and which were lodged immediately behind it. The bladder, which was greatly thickened, was filled with mucus, and contained ſix calculi. Behind the bladder was a maſs of thickened parts connecting it with the rectum, and a communication between the bladder and rectum large enough to admit a bougie.

